# Association of immunohistochemical markers with premalignancy in Gonadal Dysgenesis

**DOI:** 10.1186/s13633-015-0010-6

**Published:** 2015-06-15

**Authors:** Bonnie McCann-Crosby, Sheila Gunn, E. O’Brian Smith, Lefkothea Karaviti, M. John Hicks

**Affiliations:** Division of Pediatric Endocrinology, Baylor College of Medicine, Texas Children’s Hospital, Houston, TX 77030 USA; Department of Pediatrics, Children’s Nutrition Research Center, Baylor College of Medicine, Houston, TX 77030 USA; Department of Pathology, Baylor College of Medicine, Texas Children’s Hospital, Houston, TX 77030 USA

**Keywords:** Gonadal dysgenesis, Germ cell tumor, Gonadectomy, Immunohistochemical markers

## Abstract

**Background:**

Gonadal dysgenesis (GD) is associated with increased risk of gonadal malignancy. Determining a patient’s risk and appropriate timing of gonadectomy is challenging, but immunohistochemical markers (IHM) may help establish the diagnosis of malignant germ cell tumors (GCT). Our objective was to identify the prevalence of specific IHM expression in patients with GD and determine if the patterns of expression can help identify malignancy versus pre-malignancy state. We evaluated the published literature using the Grading of Recommendation, Assessment, Development, and Evaluation (GRADE) system to provide recommendations on the predictive role of IHM in the detection of germ cell malignancy.

**Methods:**

The data for this retrospective study included karyotype, gonadal location, external masculinization score, age at time of gonadectomy or biopsy, microscopic description and diagnosis of gonadal tissue, and immunohistochemical staining, including octamer binding transcription factor (OCT) 3/4, placental-like alkaline phosphatase (PLAP), β-catenin, alpha-fetoprotein (AFP), and stem cell factor receptor CD117 (c-KIT). Patients with complete or partial GD who had undergone gonadectomy or gonadal tissue biopsy were included.

**Results:**

The study included 26 patients with GD, 3 of whom had evidence of GCT (11.5 %, gonadoblastoma, dysgerminoma): 2 had Swyer syndrome, 1 had 46,XY partial GD. One patient with XY partial GD had gonadoblastoma-like tissue. All 4 patients (15 %) had strong expressions of 4 tumor markers (OCT 3/4, PLAP, β-catenin, CD117), as did 5 other patients (19 %, ages 2–14 months) without GCT: 4 had XY GD, 1 had 46,XX GD. β-catenin was expressed in 96 % of patients in a cytoplasmic pattern, CD117 in 78 %, OCT 3/4 in 55 %, PLAP in 37 %, and AFP in 1 patient (4 %). Tumor marker expression was not specific for ruling out malignancy in patients <1 year.

**Conclusions:**

In patients older than 1 year, expression of all three markers (OCT 3/4, PLAP, CD117) may be instrumental in the decision-making process for gonadectomy, even in the absence of overt germ cell malignancy. Our literature review suggests that OCT 3/4 expression is most helpful in predicting risk of malignancy. Additional criteria are needed to stratify risk in patients younger than 1 year of age, as these markers are not reliable in that age group.

## Background

Gonadal dysgenesis (GD), a condition with interrupted gonadal development leading to gonadal dysfunction, is a subset of disorders of sexual differentiation (DSD). Dysgenetic gonads are characterized by varying degrees of immaturity or dysfunction which can present with a wide range of genital ambiguity. Depending on the gonadal morphology, GD can be classified as either complete (CGD) or partial (PGD) [1]. CGD is characterized by a lack of testicular development, presenting as phenotypic females with bilateral streak gonads. In contrast, PGD is characterized by partial testicular development and often presents with ambiguous genitalia. The gonadal histology in PGD typically consists of hypoplastic testicular tubules intermixed with areas of ovarian-like stroma [2]. Patients with GD who have Y-chromosome material are at increased risk for the development of type II germ cell tumors (GCT), including dysgerminoma (DG) and seminoma arising from their precursor lesions gonadoblastoma (GB) and carcinoma *in situ* (CIS)/intratubular germ cell neoplasia unclassified (ITGCNU), respectively [3]. Early gonadectomy has been recommended for patients with GD who have a Y-chromosome component to prevent the development of malignancy, but recommendations for the timing of the gonadectomy remain controversial. Although gonadectomy is effective in preventing gonadal malignancy, it leads to infertility and requires a lifelong commitment to taking hormone-replacement therapy. Guidelines for a more conservative approach than exclusively performing gonadectomy are lacking.

Determining a patient’s risk for development of gonadal malignancy is necessary in the decision-making process for performing a gonadectomy. Certain factors, including the underlying etiology of the GD, location of the gonads, degree of virilization, and certain tumor marker expression in gonadal tissue, are known to influence the risk of developing gonadal malignancy. The risk of developing gonadal malignancy in patients with 46, XY complete GD (CGD; i.e., Swyer syndrome) has been reported to be 15-45 %, whereas the risk is reported to be 15-40 % in patients with partial GD (PGD) who have 45,X/46,XY and other chromosomal variants [4–7]. Gonads that are located intra-abdominally are at higher risk of developing tumors compared to those that are located in the scrotum. Studies also have shown that a correlation exists between the degree of virilization of the external genitalia and the risk of developing gonadal malignancy, with the lowest risk seen in normally virilized males and the highest risk in patients with ambiguous genitalia, reflective of the degree of testicular function [8].

Immunohistochemical markers including octamer binding transcription factor (OCT) 3/4, stem cell factor receptor CD117 (C-KIT), testis-specific protein on the Y chromosome (TSPY) gene, alpha-fetoprotein (AFP), placental-like alkaline phosphatase (PLAP), and β-catenin have been established as markers of germ cell malignancy. Several studies have investigated the use of these markers to assess for early neoplastic changes. OCT 3/4 is a transcription factor that is essential for the maintenance of pluripotentiality of embryonic stem cells and primordial germ cells [9]. Abnormal regulation of OCT 3/4 leads to inappropriate cell survival and has been shown to be present in precursor cells of type II GCTs. β-catenin is involved in regulating cell differentiation and may interact with OCT 3/4 in the transformation of GB into seminoma/DG [10]. CD 117 is the receptor for stem cell factor (SCF), and is responsible for proper migration of primordial germ cells during development. It is present only in immature germ cells and is highly expressed in early stages of germ cell development [11]. Placental-like alkaline phosphatase (PLAP) is a marker for primordial germ cells and is present in CIS and GB [12]. The TSPY gene is thought to act as an oncogene in the context of a dysgenetic gonad, leading to the development of GB [13]. AFP is a well-known marker for GCTs with yolk sac differentiation [14].

The objective of our study was to analyze the premalignant and malignant gonadal pathology findings in patients with GD and to determine the prevalence of specific immunohistochemical marker expressions. Our findings prompted us to evaluate the literature using the Grading of Recommendation, Assessment, Development, and Evaluation (GRADE) system on risk factors that are predictive of malignancy, as this is a major concern for pediatric endocrinologists and urologists when they must decide which patients require gonadectomy, and which may benefit from a more conservative approach with retention of the gonads.

## Methods

The protocol for this study was approved by Institutional Review Board at Baylor College of Medicine, Houston, Texas.

### Tissue collection and clinical data

Gonadectomy or gonadal biopsy samples from 26 patients with GD were retrieved from the archives of the pathology department at Texas Children’s Hospital, Houston, Texas. All samples were reviewed by M.J.H., an American Board of Pathology (Tampa, FL, USA) certified anatomic pathologist experienced in germ cell malignancy and gonadal histology, in order to confirm the diagnosis and determine if adequate tissue from the lesions was available for immunohistochemical evaluation. A patient was considered to have XY GD if there was an XY karyotype and histologic evidence of deficient testicular development. A patient was considered to have XX GD if there was an XX karyotype and histologic evidence of testicular development and ambiguous genitalia. Patients who had complete absence of testicular tissue with a female phenotype were considered to have CGD, whereas patients who had some testicular tissue and ambiguous genitalia were considered to have PGD. Additional clinical data were obtained retrospectively from the medical records and included the patients’ karyotype, age at time of gonadectomy or gonadal biopsy, location of gonads, sex assignment, and the external masculinization score (EMS). The EMS is an objective measure of the degree of virilization of the genitalia and takes into account individual features including the presence or absence of scrotal fusion, location of the urethral meatus, penile length, and the location of each gonad [15]. The scores range from 0–12, with a score of 0 representing a phenotypic female and a score of 12 representing a normally virilized male. Patients with lack of detailed external phenotype descriptions or insufficient gonadal tissues for staining and review were excluded.

### Immunohistochemical staining

Tissue sections were prepared from formalin-fixed embedded paraffin tissue blocks for hematoxylin-eosin (H&E) staining for routine microscopic examination and for immunohistochemical staining. All routine H&E and immunohistochemical staining were performed in an anatomic pathology laboratory certified by the College of American Pathologists (CAP) (Northfield, IL, USA), using procedures certified by the CAP with appropriate positive and negative controls and using automated H&E (Leica Microsystems, Inc., Buffalo, IL, USA) and immunohistochemical (Leica Bond III Automated Immunohistochemical and *In Situ* Hybridization Biosystem, Leica Microsystems) staining systems that have undergone inspection by the CAP. Automated immunohistochemical staining was performed using proprietary citrate-buffered and surfactant reagents (Novocastra Bond Epitope Retrieval System 1, Leica Microsystems, Inc.) and proprietary antibody kits (Novacastra-Leica Microsystems, Inc.) titrated for OCT 3/4 (clone: NCL-L-OCT3/4), PLAP (clone: NCL-L-PLAP-8A9), β-catenin (clone: NCL-L-B-CAT), CD117 (clone: (c-kit) (clone: YR145 Rab)), and AFP (clone: NCL-L-AFP). Appropriate positive and negative controls for each antibody were utilized. All tissue sectioning, routine H&E staining, and immunohistochemical staining were performed by histotechnologists certified by the American Society of Clinical Pathology (ASCP) (Chicago, IL, USA).

Microscopic examinations of the immunohistochemical staining with each antibody were reviewed concurrently by two examiners (MJH, BMC) in a blinded fashion. The tissue sections from each specimen were evaluated based upon strength of immunoreactivity (negative = 0; weak +; strong ++), origin of tissue immunoreacting (ovarian, testicular), and the cytologic component immunoreacting (cytoplasmic or nuclear).

### Statistical analysis

The prevalence of germ cell neoplasia was calculated for each subgroup of GD (XY CGD, XY PGD, and XX GD). Sensitivity and specificity for each immunohistochemical marker were calculated with 95 % confidence intervals. The positive predictive value for each immunohistochemical marker was calculated using Bayes rule, given the reported prevalence of germ cell neoplasia in GD as 15-40 %.

### Evaluation of the literature

We identified a clinically relevant question to be answered from the evidence for the management of patients with GD:

*In patients with gonadal dysgenesis, is there a predictive role of immunohistochemical markers in the detection of germ cell malignancy?*

We searched databases for research-based articles on pediatric patients with GD and immunohistochemical staining. The databases included Pub Med, Cochrane Collaboration, and Google Scholar. We included only articles published in English and studies that included more than five patients. Specific keywords and terms used included: immunohistochemistry, XY gonadal dysgenesis, germ cell tumor, GB, and DG. The GRADE system was used to evaluate the literature and provide recommendations [16]. The quality of the evidence was evaluated as “very-low quality,” “low quality,” “moderate quality,” or “high quality.” The recommendations provided were either “strong” or “weak”.

## Results

**Fig. 1:**
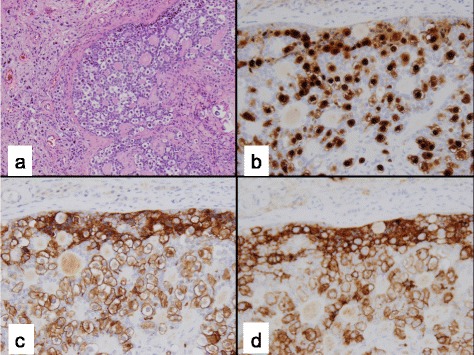
Gonadoblastoma arising within Gonadal Dysgenesis: **a** Gonadoblastoma with large germ cells with vesicular nuclei, prominent nucleoli and abundant cytoplasm and hyaline globules infiltrating adjacent gonad (H&E stain); **b** Nuclear immunoreactivity with OCT3/4; **c** CD117 (c-Kit) cytoplasmic immunoreactivity; **d** Placental alkaline phosphatase (PLAP) cytoplasmic immunoreactivity

**Fig. 2:**
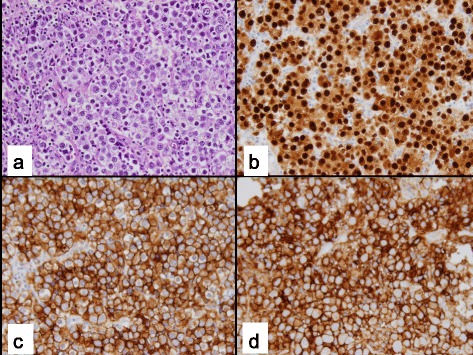
Dysgerminoma arising within Gonadal Dysgenesis: **a** Dysgerminoma with sheets of round large uniform tumor cells with granular cytoplasm and with lymphocytes in background (H&E stain); **b** Nuclear immunoreactivity with OCT3/4; **c** CD117 (c-Kit) cytoplasmic immunoreactivity; **d** Placental alkaline phosphatase (PLAP) cytoplasmic immunoreactivity

**Fig. 3:**
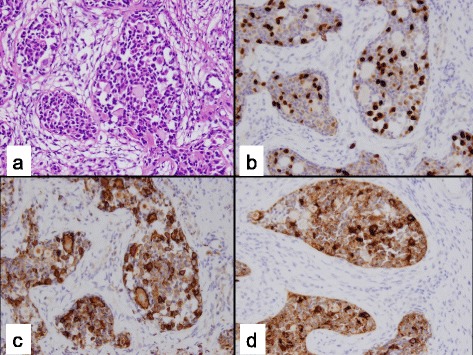
*In Situ* gonadoblastoma arising within Gonadal Dysgenesis: **a** Several noninvasive gonadoblastoma-like nests comprised of large germ cells with vesicular nuclei, prominent nucleoli, and abundant cytoplasm and hyaline globules (H&E stain); **b** Nuclear immunoreactivity with OCT3/4; **c** CD117 (c-Kit) cytoplasmic immunoreactivity; **d** Placental alkaline phosphatase (PLAP) cytoplasmic immunoreactivity

The clinical data for the 26 cases included in this study are shown in Table 1. Patient 14 had a right gonadectomy at age 12 months and a left testicular biopsy at 33 months, thus a total of 27 gonadal samples were reviewed in this study. Twenty patients had XY PGD, four patients had XX PGD, and two patients had XY CGD. The age at the time of gonadectomy or gonadal biopsy ranged from 2 months to 18 years (median, 20 months). Three patients (11.5 %) had evidence of GCT, two of whom had XY CGD and one who had XY PGD. Both of the XY CGD patients had both a GB (Fig. 1) and DG (Fig. 2), whereas the patient with XY PGD had only a GB. An additional patient with XY PGD had tissue resembling a GB (Fig. 3) and was classified as having *in-situ* neoplasia.

**Table 1:** Clinical characteristics and immunohistochemical staining results

Patient	Age	Diagnosis	Sex assignment (M/F)	Karyotype	Gonadal location	EMS out of 12	Malignancy	Gonadal tissue type	OCT 3/4	PLAP	Β-Catenin	AFP	CD117
1	6 y	XY PGD	F	45,X[16]/46,X + mar[4], SRY positive	Abdomen	1	No	Streak	-	-	++C	-	+C
2	11 y	XY PGD	F	45,X/46,XY	Abdomen	1	No	Streak	-	-	++C	-	+C
3	12 y	XY PGD	F	45,X/46,XY	Abdomen	1	No	Streak	-	-	++C	-	-
4	16 y	XY PGD	F	45,X[11]/46, X, idic (Y) (q11.21)	Abdomen	1	No	Streak	-	-	++N	-	-
5	7 m	XY PGD	M	45, X/46, XY	L- abdomen	8.5	No	Streak	-	-	++C	-	-
					R- scrotum								
6	7 m	XY PGD	M	45, X/46, XY	L-Scrotum	11.5	No	Streak	-	-	++C	-	-
					R-abdomen								
7	4 m	XX PGD	M	46, XX	Inguinal	3	No	O/T	+C, T & O	-	++C, T & O	-	+C, T
													++C, O
8	6 m	XX PGD	M	46, XX	Inguinal	4	No	O/T	+C, T & O	-	++C, T & O	-	+C, T
													++C, O
9	2 y	XX PGD	M	46, XX	Inguinal	5	No	O/T	+C, T & O	-	-T	-	-T
											++C, O		++C, O
10	5 m	XY PGD	M	46, XX/46, XXY	L-scrotum	6	No	O/T	++N, T	++C, T	++C, T & O	-	++C, T & O
					R- abdomen								
									+C, O	-O			
11	8 m	XY PGD	M	46, XY with mosaicism for 45, X/46, XY in 40 % of cells	L- abdomen R- scrotum	6.5	No	O/T	-	-	++C	++C	+C
12	9 y	XY PGD	M	46, XY/47, XXY/45,X	Abdomen	8	No	O/T	-	-	++C	-	-T
													+C, O
13	14 m	XX PGD	M	46, XX	L- inguinal	8.5	No	O/T	++N	++C	++C	-	++C
					R- scrotum								
14a	12 m	XY PGD	M	45, X/46, XY	R abdomen	8	No	O/T	++N	++C	++C	-	++C
14b	33 m	XY PGD	M	45, X/46, XY	L-scrotal	8	No	Dysgenetic Testis	+N	-	++C	-	+C
15	15 y	XY PGD	F	46, XY and gain of chrom 16p11.2	Inguinal	2	No	Dysgenetic Testis	-	-	+C	-	-
16	2 m	XY PGD	F	45, X/46, XY	Abdomen	4	No	Dysgenetic Testis	++N	++C	++C	-	++C
17	22 m	XY PGD	F	45, X/46, XY	Inguinal	5	No	Dysgenetic Testis	++N	++C	++C	-	+C
18	4 y	XY PGD	F	46, XY with gain of chrom 2q14.1	Inguinal	5	No	Dysgenetic Testis	-	-	++C	-	-
Patient	Age	Diagnosis	Sex assignment (M/F)	Karyotype	Gonadal location	EMS out of 12	Malignancy	Gonadal tissue type	OCT 3/4	PLAP	Β-Catenin	AFP	CD117
19	16 y	XY PGD	F	46, XY	Inguinal	5	No	Dysgenetic Testis	+C	-	++C	-	+C
20	11 m	XY PGD	M	46, XY	Inguinal	6	No	Dysgenetic Testis	-	-	-	-	-
21	6 m	XY PGD	M	46, XY	L-scrotum	9	No	Dysgenetic Testis	++N	++C	++C	-	++C
					R- abdomen								
22	20 m	XY PGD	M	45, X/46, XY	Inguinal	9	No	Dysgenetic Testis	-	-	++C	-	-
23	7 m	XY PGD	F	46, XY, t(11;16)(q22.1;q12.2)	Abdomen	1	GB-like	GB-like arising from immature testicular tissue	++N	++C	++C	-	++C
24	11 m	XY PGD	M	46, XY	L- abdomen	9.5	L gonad- GB	GB arising from streak-like ovarian tissue	++N	++C	++C	-	++C
					R- scrotum								
25	17 y	XY CGD	F	46, XY	Abdomen	1	GB with DG	DG and GB arising from steak gonad with Ovarian stroma	++N	++C	++C	-	++C
26	18 y	XY CGD	F	46, XY	Abdomen	1	R ovary- GB & DG	GB and DG arising from streak gonad with Ovarian stroma	++N	++C	++C	-	++C
							L ovary- GB						

*PGD* Partial Gonadal Dysgenesis, *CGD* Complete Gonadal Dysgenesis, *O/T* Ovarian and Testicular Components, *DG* Dysgerminoma, *GB* Gondadoblastoma, *-* negative staining, + weakly positive, ++ strongly positive, *C* cytoplasm, *N* nuclei, *T* testicular tissue, *O* ovarian tissue

The immunohistochemical findings are summarized in Table 1 according to the type of gonadal tissue that was encountered (streak gonad, ovarian and testicular components, testicular tissue, GB-like, GB, and DG). The three patients with a GCT and the one patient with GB-like tissue all showed strong expression of four tumor markers (OCT 3/4, PLAP, β-catenin, and CD117). Five additional patients (patients 10, 13, 14a, 16, and 21) who did not have evidence of GCT on microscopy also showed strong expression of the same four markers. Four of these five additional patients (age ranges, 2–12 months) had XY PGD and one (aged 14 months) had XX PGD. The overall expression of the immunohistochemical markers from all cases was as follows: β-catenin in 96 % of total samples, CD117 in 78 %, OCT 3/4 in 55 %, PLAP in 37 %, and AFP in 4 %.

The sensitivity, specificity, and positive predictive value (given the prevalence of gonadal malignancy in GD as reported in the literature at 15-40 %) were calculated for each immunohistochemical marker individually, as well as the combination of OCT 3/4, PLAP, and CD117 and are shown in Table 2. Positive staining for all three markers (OCT 3/4, PLAP, and CD117) as well as positive staining alone for PLAP showed the highest sensitivity (100 %, 95 % CI 40.2-100 %), specificity (73.9 %, 95 % CI 51–89.7 %), and positive predictive value (40.3-71.9 %).

**Table 2:** Sensitivity, specificity, and positive predictive value for tumor markers (n = 26)

Tumor markers	Sensitivity (95 % CI)	Specificity (95 % CI)	Positive predictive value given prevalence of gonadal malignancy as 15-40 %
OCT 3/4, PLAP, and CD117 combined	100 % (40.2-100 %)	73.9 (51.6-89.7 %)	40.3-71.9 %
OCT 3/4	100 % (40.2-100 %)	52.2 % (30.6-73.1 %)	26.9-58.2 %
PLAP	100 % (40.2-100 %)	73.9 % (51.6-89.7 %)	40.3-71.9 %
Β-catenin	100 % (40.2-100 %)	4.4 % (0.7-22.0 %)	15.6-41.1 %
CD117	100 % (40.2-100 %)	34.8 % (16.4-57.3 %)	21.3-50.6 %
AFP	0 % (0-59.8 %)	95.7 % (78.0-99.3 %)	0 %

Calculations based on the total case number of 26

With respect to gonadal location, all samples with germ cell neoplasia originated from abdominal gonads. Three of the four patients with germ cell neoplasia were phenotypic females with an EMS score of 1 (Patient 23 had clitoromegaly and a single introitus which is not accounted for using the EMS scoring system as there is no additional score for clitoromegaly alone), whereas one patient with XY PGD was phenotypically male and had an EMS score of 9.5.

### Literature review: evidence and recommendations

*In patients with GD, is there a predictive role of immunohistochemical markers in the detection of germ cell malignancy?*

### Evidence

Seven observational studies published between 2001 and 2013 were identified that assisted in answering this question [10, 17–22]. The GRADE tool was used to evaluate the evidence and provide recommendations. These studies are summarized in Table 3.

**Table 3:** GRADE evaluation of literature for immunohistochemical markers that can be used to determine high risk of malignancy in GD

Quality assessment			Summary of findings		Quality
Author	Study design and objective	Design limitations	Sample	Results/Conclusions	
		Inconsistency of results			
		Indirectness of evidence			
Palma (2013)	Retrospective Observational Study	Insufficient sample size	18 gonadal samples from 15 pediatric patients with 45,X/46,XY PGD	1 patient had GB, 1 patient had DG	Low
	To determine whether OCT 3/4 and β-Catenin are expressed in dysgenetic gonads before GB development and whether TSPY participates in malignant invasive behavior	No inconsistencies	Ages not specified	14/18 samples stained + for OCT 3/4	
		Head-to-Head comparison in correct population		Only 3 samples stained + for β-catenin	
				-Seen in Dysgenetic testes, UGT, GB, and DG, not + in streak tissue or mature germ cells	
				Tissue expressing OCT 3/4 and TSPY is associated with a high risk for GB development.	
				Suggest that β-catenin is not involved in dysgenetic gonad progression to GB, participates after GB is established.	
Barros (2011)	Retrospective Observational Study	Insufficient sample size	32 gonadal samples from 16 patients with Turner Syndrome and Y chromosome material	19 % had + nuclear OCT4 staining, suggesting the presence of germ cell tumor cells (likely GB or CIS)	Low
	To investigate the frequency of gonadal tumors among patients with Turner syndrome and Y-chromosome material	No inconsistencies	Ages 8–18 yrs.	OCT4 immunohistochemistry is more sensitive than conventional H&E staining to indicate the risk of development of germ cell tumors in TS patients	
		Head-to-Head comparison in correct population			
Palma (2008)	Retrospective Observational Study	Insufficient sample size	7 patients with PGD and GB	OCT 3/4 was + in the nuclei of immature germ cells in GB	Low
	To evaluate the participation of β-catenin and OCT 3/4 in the oncogenic pathways involved in the transformation of GB into seminoma/DG	No inconsistencies	Ages 2–33 m	Β-catenin was overexpressed in immature germ cells in GB	
		Head-to-Head comparison in correct population		Β-catenin and OCT 3/4 co-localized in immature germ cells in GB nests in all cases	
				The proliferation of immature germ cells in GB may be due to an interaction between OCT 3/4 and accumulated β-Catenin in the nuclei of the immature germ cells	
Cools (2006)	Retrospective Observational Study	Insufficient sample size	60 gonadal samples from 43 patients with GD	Incidence of GCTs was 35 %	Low
	To define the histological origin of GB, allowing the identification of high-risk patients	No inconsistencies	Ages 1 m-25 yrs.	Germ cells within GB were + for OCT 3/4, c-KIT, PLAP, and TSPY	
		Head-to-Head comparison in correct population		In UGT found adjacent to GB, OCT 3/4, PLAP, and c-KIT + germ cells were found	
				A gonadal biopsy revealing the presence of UGT with OCT 3/4 + cells on the basal lamina contains high risk for GCT and should lead to gonadectomy.	
Quality assessment			Summary of findings		Quality
Author	Study design and objective	Design limitations	Sample	Results/Conclusions	
		Inconsistency of Results			
		Indirectness of Evidence			
Cools (2005)	Retrospective Observational Study	Insufficient sample size	58 gonadal samples from 30 patients with undervirilization syndromes	OCT 3/4 was found in all patients <9 m of age	Low
	To distinguish germ cells with maturation delay from those with CIS	Different populations (not looking specifically at GD patients)	Ages 1 m-23 yrs.	-In young patients and controls, OCT 3/4 + cells were found centrally in the tubule	
				-In 3 older patients with CIS, OCT 3/4 + cells were found along the basal lamina	
				Expression of PLAP and c-KIT was similar to OCT 3/4, but less consistent	
				The presence of germ cells + for OCT 3/4, PLAP, or c-KIT in patients < 1 yr is in accordance with expected maturation delay and is insufficient for the diagnosis of CIS.	
				The location of OCT 3/4 positive cells is important in differentiating between CIS and maturation delay	
Kersemaekers (2005)	Retrospective Observational Study	Insufficient sample size	6 gonads from 5 patients with GD containing GB	4 patients had DG arising from GB	Low
	To investigate the pathogenesis of GB and evaluate its relationship to CIS.	No inconsistencies			
		Head-to-Head comparison in correct population	Ages 14–21 yrs.	c-KIT was the least consistent marker	
				PLAP was + in all GBs and adjacent invasive components.	
				Most of the tumor cells in invasive DG were weakly + or - for PLAP.	
				OCT 3/4 was + in all GBs and DGs	
				Seen in more immature cells, not mature cells	
				The development of an invasive germ cell tumor seems to involve selection and clonal expansion of an immature germ cell + for OCT 3/4 and TSPY	
Slowikowska-Hilczer (2001)	Retrospective Observational Study	Insufficient sample size	23 patients with XY GD	On the basis of PLAP expression, CIS was detected in 10 cases (43.5 %) with GD.	Low
			Ages 3 m-7 yrs.	GB was found in 4 cases of GD and DG was found in 1 patient with GD (17 years old)	
		No inconsistencies			
	To investigate the appearance of CIS in patients with 46,XY testicular dysgenesis in different ages and in adult patients from other groups				
		Head-to-Head comparison in correct population			
				Results showed a high prevalence of CIS in XY GD, indicating the importance of early histopathological evaluation of the gonads in these patients	

Among all the immunohistochemical markers evaluated, OCT 3/4 had the most consistent staining intensity for GB and DG, with PLAP and CD 117 showing less consistent expression. Several studies have shown expression of OCT 3/4, PLAP, and CD 117 in undifferentiated gonadal tissue adjacent to GB nests. Multiple studies have suggested that OCT 3/4 -positive immature germ cells in dysgenetic testes or undifferentiated gonadal tissue are at high risk for development of GB [19–21].

The location of OCT 3/4-positive cells has been shown to be important in differentiating between an *in-situ* neoplasia and maturation delay. In one study, all children younger than 9 months old had OCT 3/4-positive cells that were found centrally in the seminiferous tubules, whereas in three older patients with CIS, OCT 3/4-positive cells were located along the basal lamina [20]. The presence of OCT 3/4-positive cells in testicular parenchyma of patients who are younger than 1 year of age is thought to be due to germ cell maturation delay and, thus, cannot be used to diagnose CIS [11].

### Recommendations

1. In patients who are older than 1 year of age, the presence of OCT 3/4-positive immature germ cells located along the basal lamina in dysgenetic testes or undifferentiated gonadal tissue confers a high risk for development of germ cell neoplasia and should lead to gonadectomy.

Evidence Quality: Low

Strength of Recommendation: Strong

2. In patients who are younger than 1 year of age, caution should be taken in interpreting positive OCT 3/4 cells, as this finding can be reflective of normal delay of germ cell maturation.

Evidence Quality: Low

Strength of Recommendation: Weak

## Discussion

We studied 27 gonadectomy and gonadal biopsy samples in 26 patients with GD to investigate pre-malignant and malignant characteristics and identify factors that might be useful in predicting the development of germ cell malignancy and, thus, guide the decision-making process for performing a gonadectomy. Our findings as compared to what has been reported in the literature are summarized in Table 4.

**Table 4:** Comparison of features between the present study and previous studies

	Present study	Previous studies
Prevalence of germ cell tumor in GD population	11.5 %	15-40 %
Age	7 m - 18 y	Wide age range at presentation. GB has been identified in cases < 1 yr of age.
Location of gonads in patients with malignancy	100 % in abdomen	Abdominal gonads have been shown to have highest risk of malignant transformation.
Degree of virilization in patients with malignancy	3 of 4 pts were phenotypic female.	Low risk: Normally virilized males
	1 pt was ambiguous.	Intermediate Risk: Mild undervirilization
		High Risk: Ambiguous genitalia
Gross pathology findings	3 pts had GB arising from streak gonad with ovarian stroma.	Low Risk: Streak gonad without germ cells, ovary, testis without immature germ cells
	1 pt had GB arising from immature testicular tissue.	High Risk: Undifferentiated gonadal tissue, dysgenetic testicle
Immunohistochemistry	All pts with GCT had strong expression of OCT 3/4, PLAP, β-catenin, and CD117	OCT 3/4, PLAP, β-catenin, and CD117 are established markers of germ cell malignancy.

The overall prevalence of GCTs found in the 26 patients with GD included in this series was 11.5 % (n = 3), with an additional patient having evidence of *in-situ* GB-like tissue. Invasive GCTs were found in 7.7 % (n = 2). Excluding the four patients with XX GD, the prevalence of GCTs becomes 13.6 %, which is slightly lower than the prevalence of 15-40 % seen in patients with XY GD as reported in previous studies. An important limitation in interpreting the prevalence of GCT in our study compared to previous studies is our small sample size. All patients in our series with a GCT had abdominal gonads, consistent with previous studies reporting the highest risk of malignancy in abdominal gonads and the lowest risk in scrotal gonads. The age of the patients in our study with *in-situ* or invasive germ cell neoplasia ranged from 7 months to 18 years, with the two older patients (ages 17 and 18 years) having invasive germ cell neoplasia. GB has been described in patients with XY GD younger than 1 year of age [23, 24].

OCT 3/4, CD117, and PLAP are established markers of germ cell malignancy, and these three markers were easily identified in all patients in our series with either *in-situ* or invasive GCTs. Whereas all the immunohistochemical markers besides AFP provided high sensitivity for GCTs, PLAP showed the highest specificity in our study. β-Catenin was identified in 96 % of the samples and, thus, was the least specific for germ cell neoplasia. Consistent with what has been seen in other studies, OCT 3/4 showed robust, nuclear staining and was easily identifiable [25]. Our data suggest that in patients with GD who are older than 1 year of age, the simultaneous expression of OCT 3/4, PLAP, and CD117 should be an indication for performing gonadectomy, as these markers are highly sensitive for germ cell malignancy and pre-invasive lesions. In patients reared as males with mild undervirilization and gonads that can be repositioned surgically into the scrotum, gonadal biopsy with staining for these markers should be considered at the time of orchidopexy to evaluate the malignant potential.

Although OCT 3/4, CD117, and PLAP are highly sensitive, their low specificity renders their use in clinical practice difficult in ruling out germ cell malignancy, particularly when evaluating patients who are younger than 1 year of age. A study looking at these markers in normal fetal testicular development showed that OCT 3/4, CD 117, and PLAP can be present in the germ cells of neonates as germ cell maturation delay or block is expected, and they are unreliable in detecting CIS/ITGCNU in very young children [11]. As seen in our study, two patients younger than 1 year of age had evidence of GB (age, 11 months) or *in-situ* GB-like tissue (age, 7 months); hence, further criteria are needed to identify patients at risk for malignancy in this age group. In these younger patients, surrogate measures including location of gonads, morphology of gonadal tissue, function of testicular tissue as determined by hormonal studies, and degree of virilization should be evaluated to determine risk for developing malignant transformation. Future outcomes studies are needed to determine the usefulness of these additional measures in determining the potential for development of a malignancy.

## Conclusions

We analyzed our data and used a systematic method to evaluate the literature to provide recommendations for the usefulness of immunohistochemical markers in detecting premalignant and malignant germ cell tumors in gonadal dysgenesis. Our findings are consistent with the literature with regards to the usefulness of OCT 3/4 as a marker that is highly reliable for germ cell malignancy and pre-invasive lesions in patients older than 1 year of age. Our data also demonstrate the usefulness of PLAP and CD117 in addition to OCT 3/4 in assessing pre-malignant potential. Additional criteria seen in both our study and the literature including abdominal gonads and undervirilization are important risk factors for germ cell malignancy in patients with XY gonadal dysgenesis. It is critical to identify each patient’s individual risk for malignancy and careful consideration is required before gonadal biopsy and/or gonadectomy are recommended. As seen in our study, patients younger than 1 year of age can present with germ cell malignancy, however, the use of immunohistochemical markers is not reliable in assessing premalignancy in this age group. Thus, to prevent unnecessary gonadectomy in these younger patients, further studies are needed to evaluate surrogate measures that can be used to predict risk of malignancy. In summary, we have set the stage for evaluation of malignancy risk and the decision-making process for gonadectomy in patients with GD using immunohistochemistry; an approach confirmed by our literature review. We have indicated that surrogate measures need to be elaborated to determine which patients require gonadal biopsy and/or gonadectomy. This paper offers a preliminary overview of the available evidence and what risk factors need to be assessed in anticipation of gonadectomy. A more standardized approach is needed for the management of these patients.

## Abbreviations

GD: Gonadal dysgenesis
CGD: Complete gonadal dysgenesis
PGD: Partial gonadal dysgenesis
MGD: Mixed gonadal dysgenesis
GB: Gonadoblastoma
DG: Dysgerminoma
CIS: Carcinoma *in situ*
TSPY: Testis-specific protein-Y
DSD: Disorders of sex development
ITGCNU: Intratubular germ cell neoplasia unclassified
GCT: Germ cell tumor
PLAP: Placental-like alkaline phosphatase
OCT (3/4): Octamer binding transcription factor
AFP: Alpha fetoprotein
SCF: Stem cell factor
EMS: External masculinization score
IHM: Immunohistochemical markers

## References

[1] Ostrer H. 46,XY Disorder of Sex Development and 46,XY Complete Gonadal Dysgenesis. In: Pagon RA, Adam MP, Ardinger HH, Wallace SE, Amemiya A, Bean LJH, Bird TD, Dolan CR, Fong CT, Smith RJH, Stephens K, editors. Seattle (WA): GeneReviews(R); 1993.

[2] Berkovitz GD, Fechner PY, Zacur HW, Rock JA, Snyder HM, Migeon CJ (1991). Clinical and pathologic spectrum of 46, XY gonadal dysgenesis: its relevance to the understanding of sex differentiation. Medicine.

[3] Verp MS, Simpson JL (1987). Abnormal sexual differentiation and neoplasia. Cancer Genet Cytogenet.

[4] Cools M, Drop SL, Wolffenbuttel KP, Oosterhuis JW, Looijenga LH (2006). Germ cell tumors in the intersex gonad: old paths, new directions, moving frontiers. Endocr Rev.

[5] Pleskacova J, Hersmus R, Oosterhuis JW, Setyawati BA, Faradz SM, Cools M (2010). Tumor risk in disorders of sex development. Sex Dev.

[6] Rocha VB, Guerra-Junior G, Marques-de-Faria AP, de Mello MP, Maciel-Guerra AT (2011). Complete gonadal dysgenesis in clinical practice: the 46, XY karyotype accounts for more than one third of cases. Fertil Steril.

[7] Michala L, Goswami D, Creighton SM, Conway GS (2008). Swyer syndrome: presentation and outcomes. BJOG.

[8] Cools M, Pleskacova J, Stoop H, Hoebeke P, Van Laecke E, Drop SL (2011). Gonadal pathology and tumor risk in relation to clinical characteristics in patients with 45, X/46, XY mosaicism. J Clin Endocrinol Metab.

[9] Nichols J, Zevnik B, Anastassiadis K, Niwa H, Klewe-Nebenius D, Chambers I (1998). Formation of pluripotent stem cells in the mammalian embryo depends on the POU transcription factor Oct4. Cell.

[10] Palma I, Pena RY, Contreras A, Ceballos-Reyes G, Coyote N, Erana L (2008). Participation of OCT3/4 and beta-catenin during dysgenetic gonadal malignant transformation. Cancer Lett.

[11] Honecker F, Stoop H, de Krijger RR, Chris Lau YF, Bokemeyer C, Looijenga LH (2004). Pathobiological implications of the expression of markers of testicular carcinoma *in situ* by fetal germ cells. J Pathol.

[12] Jacobsen GK, Norgaard-Pedersen B (1984). Placental alkaline phosphatase in testicular germ cell tumours and in carcinoma-*in-situ* of the testis. An immunohistochemical study. Acta pathologica, microbiologica, et immunologica Scandinavica Section A. Pathology.

[13] Li Y, Vilain E, Conte F, Rajpert-De Meyts E, Lau YF (2007). Testis-specific protein Y-encoded gene is expressed in early and late stages of gonadoblastoma and testicular carcinoma *in situ*. Urol Oncol.

[14] Talerman A, Haije WG, Baggerman L (1978). Serum alphafetoprotein (AFP) in diagnosis and management of endodermal sinus (yolk sac) tumor and mixed germ cell tumor of the ovary. Cancer.

[15] Ahmed SF, Khwaja O, Hughes IA (2000). The role of a clinical score in the assessment of ambiguous genitalia. BJU Int.

[16] Atkins D, Best D, Briss PA, Eccles M, Falck-Ytter Y, Flottorp S (2004). Grading quality of evidence and strength of recommendations. BMJ.

[17] Palma I, Garibay N, Pena-Yolanda R, Contreras A, Raya A, Dominguez C (2013). Utility of OCT3/4, TSPY and beta-catenin as biological markers for gonadoblastoma formation and malignant germ cell tumor development in dysgenetic gonads. Dis Markers.

[18] Barros BA, Moraes SG, Coeli FB, Assumpcao JG, De Mello MP, Maciel-Guerra AT (2011). OCT4 immunohistochemistry may be necessary to identify the real risk of gonadal tumors in patients with Turner syndrome and Y chromosome sequences. Hum Reprod.

[19] Cools M, Stoop H, Kersemaekers AM, Drop SL, Wolffenbuttel KP, Bourguignon JP (2006). Gonadoblastoma arising in undifferentiated gonadal tissue within dysgenetic gonads. J Clin Endocrinol Metab.

[20] Cools M, van Aerde K, Kersemaekers AM, Boter M, Drop SL, Wolffenbuttel KP (2005). Morphological and immunohistochemical differences between gonadal maturation delay and early germ cell neoplasia in patients with undervirilization syndromes. J Clin Endocrinol Metab.

[21] Kersemaekers AM, Honecker F, Stoop H, Cools M, Molier M, Wolffenbuttel K (2005). Identification of germ cells at risk for neoplastic transformation in gonadoblastoma: an immunohistochemical study for OCT3/4 and TSPY. Hum Pathol.

[22] Slowikowska-Hilczer J, Walczak-Jedrzejowska R, Kula K (2001). Immunohistochemical diagnosis of preinvasive germ cell cancer of the testis. Folia Histochem Cytobiol.

[23] Dumic M, Jukic S, Batinica S, Ille J, Filipovic-Grcic B (1993). Bilateral gonadoblastoma in a 9-month-old infant with 46, XY gonadal dysgenesis. J Endocrinol Invest.

[24] Haddad NG, Walvoord EC, Cain MP, Davis MM (2003). Seminoma and a gonadoblastoma in an infant with mixed gonadal dysgenesis. J Pediatr.

[25] de Jong J, Stoop H, Dohle GR, Bangma CH, Kliffen M, van Esser JW (2005). Diagnostic value of OCT3/4 for pre-invasive and invasive testicular germ cell tumours. J Pathol.

